# Microbial aerosol liberation from soiled textiles isolated during routine residuals handling in a modern health care setting

**DOI:** 10.1186/s40168-015-0132-3

**Published:** 2015-12-09

**Authors:** Alina Handorean, Charles E. Robertson, J. Kirk Harris, Daniel Frank, Natalie Hull, Cassandra Kotter, Mark J. Stevens, Darrel Baumgardner, Norman R. Pace, Mark Hernandez

**Affiliations:** Department of Civil, Environmental and Architectural Engineering, University of Colorado, Boulder, USA; Anchutz Medical Center University of Colorado, Denver, USA; Droplet Measurement Technologies, Boulder, Colorado USA; Department of Molecular Cellular and Developmental Biology, University of Colorado, Boulder, USA

## Abstract

**Background:**

A wide variety of specialty textiles are used in health care settings for bedding, clothing, and privacy. The ability of textiles to host or otherwise sequester microbes has been well documented; however, their reciprocal potential for liberating airborne bacteria remains poorly characterized. In response, a multi-season survey of bacterial bioaerosols was conducted in the origin and terminus of residual paths which are specifically designed to isolate soiled hospital textiles as they are moved to laundering. This survey used conventional optical particle counting which incorporated multi-channel fluorescence in conjunction with molecular phylogenetic analyses to characterize the bioaerosols liberated during soiled textile storage—immediately before and after the occupation of a modern hospital. Although outfitted with a HEPA filtration system, the number of airborne particles presenting fluorescing optical signatures consistent with airborne bacteria and fungi significantly increased in textile holding rooms soon after the hospital’s commissioning, even though these isolated residual areas rarely host personnel. The bioaerosol liberated during textile storage was characterized using Illumina MiSeq sequencing of bacterial 16S ribosomal ribonucleic acid (rRNA) genes. Gene copies recovered by quantitative PCR from aerosol collected in co-located impingers were consistent with fluorescence gated optical particle counting.

**Results:**

The relative abundance patterns of proximal bacterial bioaerosol were such that the air in the origin and terminus of textile storage rooms could not be differentiated once the hospital began processing soiled linens. Genes from microbes typically associating with human skin, feces, and hair—*Staphylococcus*, *Propionibacteria*, *Corynebacteria*, *Lactobacillus*, and *Streptococcus* spp.—dominated the aerosol abundance profiles in textile holding rooms, which were generally far less diverse than communities recovered from surfaces in patient rooms.

**Conclusions:**

These results suggest that aerosol partitioning from the routine handling of soiled textiles can contribute to airborne exposures in the health care environment.

## Background

Hospitals and satellite care clinics occupy a wide variety of different building types, which collectively host millions of patients and staff annually, yet the range of bioaerosol loads in these settings remains unknown. With the conditions experienced in this important building sector, concerns regarding human exposure to bioaerosols—as well as the fomites they can interchange with—have been raised among the public, health care workers, and the regulatory sector.

Based on conventional monitoring methods, government agencies and professional societies have published guidelines suggesting acceptable ranges for operation hospital/clinical building HVAC systems [1], as well as best practices for handling contaminated textiles and other residuals (i.e., regulated medical wastes) in these unique indoor environments [2]. While the association of infectious microbes with a wide variety of hospital materials has been indicated by culture-based techniques, the advent and affordability of real-time optical monitoring, together with established molecular techniques, have precipitated new efforts to determine indoor bioaerosol and fomite liberation potential from health care workers’ activities. This includes bioaerosols associating with common textiles (e.g., uniforms, pajamas, and linens) and other medical residuals as they are routinely collected and pass through hospitals. In this regard, what guidelines that do exist remain qualitative: it is clear that the in situ dispersal of bioaerosols directly resulting from health care workers’ activities with textile and other residual management practices may be significant but remains understudied with modern characterization tools.

Like many studies considering nosocomial source tracking, the literature in this arena is tenuous and limited in its interpretive power where juxtaposed to recent forensic microbiology advances, which can now be applied to the health care environment. Most reports investigating the nosocomial potential associated with health care service textiles remain based on conventional culture-based investigations. Since typically less than 1 % of microbes can be recovered by culture from any environment [3], ecological observations from many culture-based studies can no longer be considered robust in this context. While results from culture-based studies are widely regarded as quantitative (colony-forming units (CFU)), the magnitude of quantitative recovery as CFU from field studies is at best an indicator and can only verify the presence of a culturable pathogen as defined by classical perspectives. Indeed, viable but not culturable (VNBC) pathogens in a wide variety of environments [4], and culture-based microbe recovery from textiles is likely no exception.

 The 1949 Lancet report "*Air Infection from Dust Liberated from Clothing"* was one of the first to recognize, isolate, and articulate aerosol pathways for pathogenic microbes to partition to the atmospheric environment from textiles in health care settings [5]. However, it was not until the late 1960s, that industrial hygienists began to experimentally address the hypothesis of microbial interchange between the indoor atmospheric environment and hospital textiles, where questions were beginning to focus on the role medical uniforms might play in facilitating the aerosol partitioning of microbes [6–8]. Several investigations on the subject ensued until comprehensive (modern) reviews emerged nearly two decades later, many with military experience as their driver. Whyte and coworkers [9] leveraged pre-Vietnam era reports on human shedding of infectious “dust” to air to determine bacterial bioaerosol emission rates from hospital and reported what would be expected when conventional clothing was worn, with a range of between 300 and 19,000 bacterial particles/min; the higher value of which is close agreement with ranges recently reported by quantitative PCR analysis of aerosol associated with clothed students in an isolated classroom setting [10].

Credible literature dating back to 1969 volumes of *The Lancet* has confirmed that *Staphylococcus aureus* and other pathogens associate with, and survive on, many different types of health care textiles and uniforms regardless of efforts to wear protective aprons or other “overgarments” [11].

Hospitals are routinely outfitted with reusable textiles which are primarily meant to serve as barriers between respective patients and the health care workers who serve them—most notably bedding (linens and pajamas) and privacy curtains. Like their garment counterparts, these textiles are constructed of a wide range of natural and synthetic materials. With respect to microbial associations with non-uniform textiles, a substantial cohort of contemporary literature also exists; some investigations in this arena, however, treat non-uniform textiles as immobile surfaces, regardless of their density or patient interface. Bedding, pillow cases, and privacy curtains have been the subject of numerous culture-based studies where potential nosocomial pathogens have been recovered by culture and by modern forensic genetics. Borkow and coworkers (2008) implicated bed linens and other non-uniform textiles as active partitioning sources, positing that “contaminated textiles might be an important source of microbes contributing to indirect contact and aerosol transmission of nosocomial-related pathogens” [12]. This report paralleled a compelling 2008 editorial to the Hospital Disinfection Society that compiled converging lines evidence suggesting pajamas and bed sheets can be a significant source of nosocomial pathogens in indoor air [13].

Some of the most striking studies demonstrating airborne nosocomial agent transfer potential from textiles using current molecular methods, includes a report by a Japanese medical team [14] confirming aerosolization of antibiotic resistant *Staphylococcus* spp. during the routine handling of bed linens. Using accepted industrial hygiene methods, air was sampled in MRSA infection wards. Airborne MRSA-containing particles were isolated in all mean aerodynamic diameter ranges between 7 and 0.65 μm and averaged between 2 and 3 μm diameter before bed making but were >5 μm during bed making activities. The number was significantly higher than otherwise identical aerosol background for as long as 15 min after bed making and MRSA was detected on many proximal surfaces. The results suggest that MRSA was recirculated in the air (in relatively high numbers) and directly associated with the movement of contaminated bed linen. Also, investigating the occurrence of airborne microbe transmission in connection with contaminated textiles (and other dry surfaces) was a British study which surveyed the Liverpool adult cystic fibrosis (CF) center [15]. This report characterized the extent of environmental contamination with an epidemic strain of *Pseudomonas aeruginosa* quantified using a PCR assay specific for the Liverpool epidemic strain (LES). While no persistent environmental reservoirs were found, LES was detected in the majority of air samples from inside patients’ rooms, the adjacent ward, proximal corridor, and the outpatient clinic. This study suggested that textiles contributed to aerosols which may have played a significant role in patient-to-patient spread of LES.

With the advent of more comprehensive attention to fomite control and remedial hand-washing campaigns came concomitant questions as to how other environmental media—namely (potable) water and indoor air—may also serve as nosocomial vectors within the health care environment [16, 17]. Driven by concern over nosocomial infection rates after the extended implementation (and enforcement) of hand-washing practices in the health care industry at large, interdisciplinary research teams have brought renewed attention to the potential for bioaerosol generated by the common perturbations and movement of contaminated textiles; whether those textiles are on humans (pajamas/uniforms), are in/on bedding, or otherwise serve as some other functional barriers (curtains/aprons). In this context, Beggs and coworkers [18] published a series of articles consistent with the notion that airborne transmission of bacteria [may] contribute significantly to hospital-acquired infections; included in this series were considerations of some textiles mediating the transport of microbes.

There are standards for the separation of clean and soiled textiles [19, 20] that are widely accepted in modern hospitals. In the USA, soiled linen handling practices are surveyed periodically by the Joint Commission on Accreditation of Health Care Organizations, which requires bagging, limits volumes and caps holding times for centralized textile collection. There are many case studies of aerobiological sampling in health care settings but those implicating textiles as a bioaerosol source, have been limited by the following factors: (i) low replicate sampling designs, (ii) the use of conventional culturing assay techniques, (iii) the use of “grab” sample paradigms, and (iv) a relatively small number of observations compared to the actual numbers of environments. Modern observations of airborne microbial numbers in health care settings are rare and only beginning to emerge in literature. With this perspective in mind, we used molecular bioaerosol surveys complimented with fluorescent-optical particle characterization to determine how moving soiled textiles can contribute to bacterial bioaerosol loading during routine residuals handling in a modern hospital.

## Methods

### Textile holding room environments

The hospital that served as the site for this demonstration study contained small, nearly cubic, “holding” rooms (c.a. 12 m^3^), which were designed to store soiled textiles on each floor prior to their systemic collection for the hospital at large; each of these holding rooms was connected by a (1 m diameter) cylindrical stainless steel gravity chute (vertical), which terminated in holding carts isolated in a basement room (c.a. 22 m^3^). With exception to the basement, the soiled linen holding rooms were passively integrated with a small wall grating (c.a. 100 cm^2^) into the greater ventilation system on each floor, of which the entirety of supply and return air was HEPA-filtered, with a design air exchange rate of 1/2 h^−1^ [21]. Aerosol samples for optical and molecular analyses were collected over 48-h periods during February 2013, following the tenant finish, but prior to patient occupation; in June 2013 (summer HVAC operation mode), several months following initial patient occupations; and then again in November 2013 (winter HVAC operation mode).

### Optical particle properties

Optical particle properties were compiled in real time by counting airborne particles in the size range of airborne bacteria and fungi that had fluorescent signatures consistent with pure cultures commonly used to model the environmental fate of bioaerosols [22]. A wideband bioaerosol sensor (WIBS v4.0) similar to that previously described by Healy and coworkers [23] collected air at 1 L/min and used dual-wavelength excitation and fluorescence detection, while simultaneously measuring characteristic optical diameter from scattered light. With the portable WIBS variant used here, fluorescence was induced by sequential exposure to UV irradiation from flashlamps filtered at 280 and 370 nm. Fluorescence emitted due to 280-nm excitation was detected in two wavebands, 310–400 nm (type a) and 420–650 nm (type b), using dedicated photomultipliers. Fluorescence emitted due to 370-nm excitation was detected between 420 and 650 nm (type c). Optical diameter was determined by light scattered from exposure to a 635-nm laser; it is reported here as an equivalent optical diameter (EOD) and defined as the diameter of a spherical particle, with a fixed refractive index (calibrated with 2.0 and 2.8 μm latex beads in air at 25–45 % RH), scattering the same light intensity as the measured (bio)aerosol. Following the annotation introduced by Perring [24], fluorescence was categorized as one of the three types, which considers intensity calibrated to a known threshold in three excitation and emission bandwidths—individually and in specific combinations. Here, we observed, classified, and counted particles based on one of three dominant fluorescence motifs, where emission was observed in a single channel (type a), concomitantly in two adjacent channels with the same excitation (types a and b), or concomitantly in all three channels with different excitation as well as emission (types a, b, and c).

Leveraging these measured quantities, the following metrics were analyzed and compiled for each particle collected, which were in the optical diameter and fluorescent property range of bacteria and fungi aerosolized in laboratory studies [25]: (i) the frequency of particles that could be segregated by discrete fluorescence signal (bandwidth) into any of the dominant types described above, (ii) the average fluorescence intensity within each bandwidth or conglomerate thereof, and (iii) the average optical diameter of each particle type. These optical properties were recovered from a minimum 10^5^ particles for each location, over approximately a 24-h period.

### Aerosol collection for phylogenetic analyses

Aerosol collection for phylogenetic analyses was co-located in each textile holding room with the fluorescence/optical particle counters. These collectors were liquid impingers modified for ultra-clean DNA recovery (OMNI 3000, InnovaPrep, Drexel, MO); details of field adaptations and capture efficiency are previously described [26]. Prior to sampling campaigns, collection cartridges were irradiated in a UV Stratalinker 1800 (Stratagene, La Jolla, CA) and filled with a filter-sterilized recovery solution that consisted of phosphate-buffered saline (137 mM NaCl, 10 mM phosphate, 2.7 mM KCl, pH 7.4) and 0.005 % Tween in DNA-free water (diethylpyrocarbonate (DEPC) treated). Cartridges which served as field blanks for PCR controls were prepared at the same time as the sample cartridges, carried during sampling, and processed in the same manner as those vessels carrying actual aerosol samples.

The air-sampling rate for genetic analyses was c.a. 220 L/min, and the sampler was operated until it collected particles from a minimum of 4.5 m^3^ of air within each of the textile holding rooms (>25 % of any given room’s volume); the collection device was carefully sterilized between stations by replacing all liquid-carrying lines with new sterile tubing and flooding the contactor along with all ports with 70 % ethanol, then allowing them to air-dry. Cleaning blank samples were taken by filling the sampler (contactor and tubing) with 5 ml DEPC-treated water, which was allowed to sit in the sampler for 5 min and then extracted with a sterile syringe from the sampling port. The cleaning blanks were filtered and extracted in the same fashion as those retaining air samples.

Immediately following collection, all of the impinger fluid was filtered through sterile 0.2-μm polycarbonate filters (Isopore; Millipore, Billerica, MA), which were then placed in DNA-free microcentrifuge tubes and shipped frozen to the laboratory, where they were stored at −80 °C until processed. Aerosol DNA was recovered by dissolving the filters in phenol, chloroform, and buffer (100 mM NaCl, 200 mM Tris-Cl [pH 8.0], 20 mM EDTA containing 5 % SDS). This extraction medium was then placed in polycarbonate centrifuge tubes including micro-zirconium beads with two volumes of buffer-saturated phenol and reciprocated cold (4C) at 2500 RPM for 2 min, and then amended with cold ethanol for precipitation of nucleic acids. Remaining DNA was concentrated by centrifugation, washed with 70 % ethanol, and re-suspended in sterile DNase/RNase-free water. This purified DNA was stored at –80 °C until further analysis.

### Amplification and sequencing

Airborne bacterial profiles were determined by broad-range amplification on an Illumina MiSeq platform to sequence 16S ribosomal ribonucleic acid (rRNA) amplicons; these were generated using broad-range PCR primers that circumscribe approximately a 300 bp of the variable V1V2 variable region of [27]. PCR products were normalized using a SequalPrep™ kit (Invitrogen, Carlsbad, CA), pooled, lyophilized, purified, and concentrated using a DNA Clean and Concentrator Kit (Zymo, Irvine, CA). Pooled amplicons were quantified using Qubit Fluorometer 2.0 (Invitrogen, Carlsbad, CA), and each pool was diluted to 4 nM and denatured with 0.2 N NaOH at room temperature. The denatured DNA was diluted to 15 pM and augmented with 25 % of PhiX DNA (manufacturer’s control) immediately prior to sequencing. Paired-end sequencing was performed with version 2.0 of the Miseq Control Software, using a 500-cycle reagent kit (v 2.0). The resulting sequences were sorted by barcodes in the paired reads with a python script and assembled using PHRAP [28, 29]. These assemblages were trimmed over a moving window of five nucleotides until their average quality met or exceeded 20. Pairs that did not assemble, or trimmed sequences with more than 1 ambiguity (any non-specific base call) or shorter than 200 bp, were discarded. Potential chimeras were identified with UCHIME (usearch6.0.203_i86linux32) [30] using the Schloss SILVA reference sequences [31] and removed from subsequent analyses. Assembled sequences were aligned and classified with SINA 1.2.11 [32] using the conglomerate of bacterial sequences in SILVA 115NR99 [33]. Assembled sequences were aligned and classified with SINA (1.2.11) using the 629,124 bacterial sequences as reference configured to yield the SILVA taxonomy.

## Results

### Particle characterizations

As judged by size segregated concentrations of total and fluorescing particle numbers, as well as fluorescing particle distributions, the airborne particle loads in the soiled linen storage rooms changed markedly in response to the hospital’s occupation. The following hallmarks describe significant changes in the airborne particle populations at the origin and terminus of the soiled linen storage, after the facility began hosting patients and processing associated textiles.

**Fig. 1:**
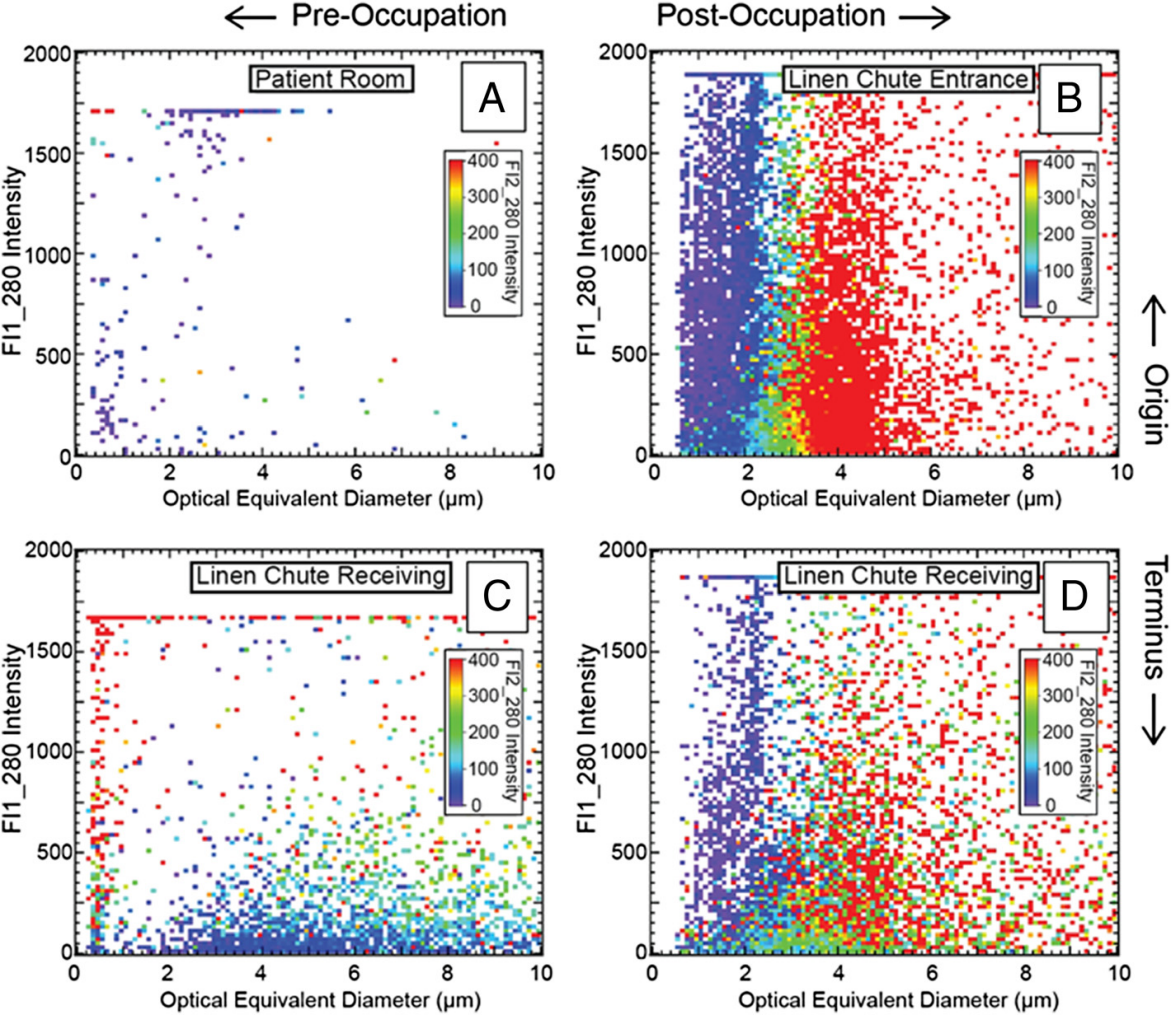
Two-channel particle fluorescence intensity distributions segregated by optical diameter as reported by a portable WIBS-4, during winter (Feb) and summer (June) sampling campaigns, 2013; 24-h composite observations. (*Top panel*: originating room) Fluorescent airborne particle monitoring pattern observed in soiled textile holding room centralized on HEPA-filtered patient floor: **a** (*top left*) 2 weeks prior to patient occupation and **b** (*top right*) 3 months after hosting patients. (*Bottom panel*: terminal room) Fluorescent airborne particle monitoring pattern observed in soiled textile receiving room isolated in basement: **c** (*bottom left*) 2 weeks prior to hospital operations and **d** (*bottom right*) 3 months after fully operational. *FL1_280* denotes relative single channel intensity scale for *type a* fluorescence; inset *FL2_280* denotes relative single channel intensity scale for *type b* fluorescence

Airborne fluorescent particle loads were significantly lower (<10^3^/m^3^) prior to hospital operations, with exception to the basement linen collection site, which hosted a threefold larger cohort of particles displaying weak fluorescence across all channels in the optical diameter (OD) range between 3 and 6 μm (Fig. 1).

The June sampling campaign (3 months following the hospital opening) revealed significant shifts in the spectral distribution presented by airborne particles in the terminal textile holding rooms, where the relative percentages of respective florescence observed in one channel (type a), two adjacent channels (types a and b), and all three channels (types a, b, and c) shifted from 12, 10, and 77 %, to a nearly even distribution of 31, 30, and 38 %. Further, the particles’ averaged fluorescence intensity was more than double than that of the pre-operational condition wherever multiple channel fluorescence was observed.

Once the hospital began moving soiled textiles, there was an obvious overlap in the patterns of fluorescing particle characteristics in the origin and terminal textile holding rooms: there were no significant differences between the averaged fluorescence intensity or sizes of the fluorescent particles present at any sampling time following the onset of hospital operations, regardless of the season. The June sampling campaign (3 months after opening) revealed a small but insignificant differences in the distribution of fluorescent signals from the airborne particles present in the origin and terminal textile holding rooms observed, where the relative percentages of florescence observed in single (type a), two (types a and b), and all three channels (types a, b, and c) shifted from 37, 44, and 19 %, respectively, at the origin, to the distribution of 31, 30, and 38 % at the terminus.

While displaying similarities in size and fluorescence spectral distributions, there were significant differences between the quantities of airborne fluorescing particles in the respective sites: the originating textile holding room had approximately twice the numbers of particles which fluoresced in the dominant channels than did the terminal textile holding room (Fig. 1).

**Fig. 2:**
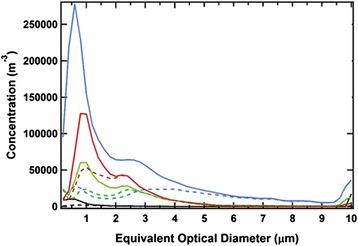
Juxtaposition of non-fluorescing (▬) and fluorescent (*■■■*) particle concentrations segregated by optical diameter as reported by a portable WIBS-4 2 weeks prior to hospital occupation (Feb 2013): patient room in HEPA-filtered floor (▬, *■■■*); terminal soiled textile storage room isolated in basement (). Particle concentrations 3 months following hospital occupation (June 2013): originating soiled textile storage room on patient floor () and terminal soiled textile receiving room isolated in basement ()

In all cases, the non-fluorescing particle loads were markedly higher than their fluorescing counter parts in the size ranges associated with airborne bacteria (0.5 μm < OD < 2 μm). The largest differences between fluorescing and non-fluorescing particle loads were in the basement terminal textile storage room just prior to the hospital’s opening (>10-fold, Fig. 2). After the first 3 months of occupation, however, the originating holding room where soiled textiles are first stored had at least twofold higher concentrations of both non-fluorescing and fluorescing particles than its terminal counterpart—a trend which was observed again in sampling campaigns executed after 3 and 8 months of hospital operations, respectively.

**Fig. 3:**
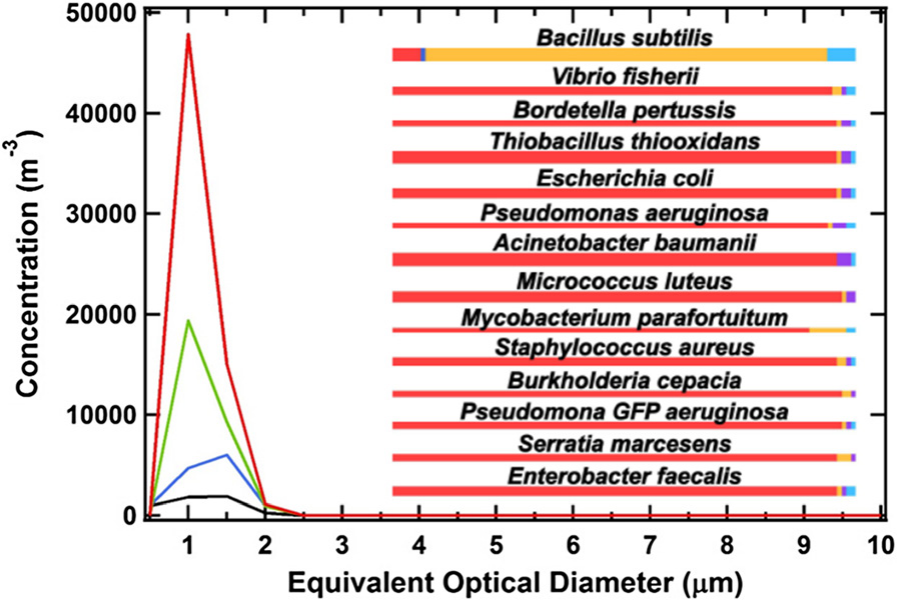
Airborne fluorescent particle concentrations segregated by optical diameter in the range of airborne bacteria (<2 μm OD) as reported by a portable WIBS-4 on a HEPA-filtered patient floor 2 weeks prior (Feb 2013) to hospital occupation: patient room (▬); proximal (originating) soiled textile storage room (). Fluorescent particle concentration 3 months following (June 2013) hospital occupation: originating soiled textile storage room on patient floor () and terminal soiled textile receiving room isolated in basement (). Fluorescent particle concentrations reported here were calibrated by, and gated to, the optical diameter, fluorescent spectral range, and specific fluorescence intensities of pure bacterial culture inset. Colored bars inset beneath bacteria culture names, represent their distribution of fluorescent type ( = type a;  = type b;  = type c); bar width represents relative optical diameter

The composite of optical diameters (OD) and fluorescent characteristics—spectral distribution and intensity—of 15 different types of common airborne bacteria were used to reference the portable WIBS for this investigation [25], and all fluorescence particle data in this size range were gated to this reference (Fig. 3). The fluorescence from particles in the OD range between 0.5 and 2.0 μm, which were not consistent with the referenced bacterial spectra used here, were respectively 37 and 40 % of the total fluorescing particles in the originating and terminal soiled textile receiving rooms; these were likely not bacteria, and thus not included in this analysis. As judged by concentrations of fluorescing particles which were in the same size and spectral range, the cohort of reference bacteria and the originating textile holding room had more than 10 times the concentration following the hospital opening than the weeks prior to this hospital floor’s occupation. Further, at all monitoring times following the hospital opening, the bacteria-referenced fluorescent particle concentrations were at least twofold higher in the originating textile holding room than those levels observed in the terminal textile holding location isolated in the basement.

### Airborne bacterial phylogenetic observations

**Fig. 4:**
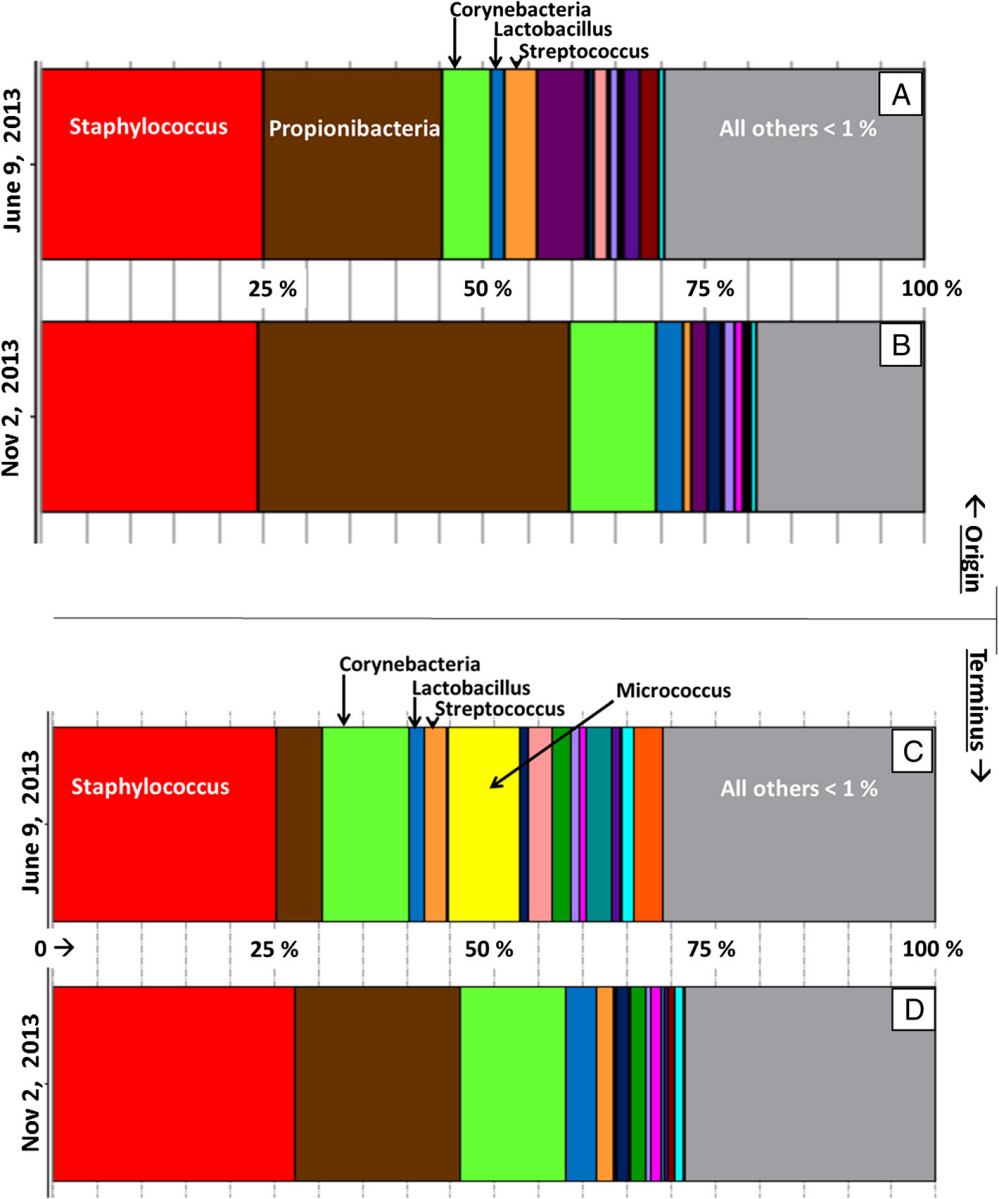
Relative abundance of 16S RNA bacteria genes recovered from 4.5 m^3^ of textile storage room air by an OMNI 3000 hi-volume impinger, during early summer (June) and early winter (November) sampling campaigns, 2013. (*Top panels*: originating room) Relative abundance pattern observed in soiled textile holding room centralized on HEPA-filtered patient floor: **a** 3 months following patient occupation and **b** 8 months following patient occupation. (*Bottom panels*: terminal room) Soiled textile receiving room isolated in basement: **c** 3 months following support staff occupation and **d** 8 months after operational. No DNA could be recovered and amplified from composite aerosol sampling prior to patient occupation (Feb 2013)

Indoor activity creates aerosol, a substantive fraction of which can be microbial. As judged by qPCR of airborne 16S RNA genes, the bacterial bioaerosol load in the textile holding rooms increased significantly following the onset of hospital operations (>5 × 10^4^/m^3^). Operational taxonomic units (OTUs) describing the airborne bacteria recovered were produced by clustering sequences with identical taxonomic assignments. Explicet v2.9.4 (www.explicet.org) [34] was used to visualize and compare the relative abundance of bacterial bioaerosol collected during the early summer and early winter, at the origin and terminus of soiled textile storage (Fig. 4). This process generated 167,556 sequences of average length 300 nt. The average number of sequences per sample was 41,889 (a minimum of 32,602 and a maximum of 50,913). The median Goods coverage score for bioaerosol libraries generated here was >99.65 %, indicating that the depth of sequencing was sufficient to describe the diversity within these samples [35, 36]. Alpha diversity was calculated at the rarefaction point of 32,950 sequences with 1000 bootstrap re-samplings (rarefied Goods coverage, *p* values via two-part analyses [37]).

None of the reagents, analytical blanks or field blanks (filled cartridges), yielded any amplifiable DNA; nor, could any 16S RNA genes be recovered from aerosol/air-filter samples at any location prior to the occupation of the hospital. Within 3 months, however, bacterial DNA could be recovered from aerosols in both textile holding rooms even though doors to these rooms remained closed as policy, and any staff intervention in these rooms is generally negligible in time frame.

As judged by recovery of airborne 16S RNA genes, the assemblages of airborne bacteria were such that the (isolated) room air at a HEPA-filtered origin and terminus of soiled textiles storage could not be differentiated following the hospital’s opening. In the order of their abundance, 16S RNA genes nearly identical to those of *Staphylococcus*, *Propionibacteria*, *Corynebacteria*, *Lactobacillus*, and *Streptococcus* spp. dominated the bioaerosol in the textile holding rooms observed, which were generally far less diverse than communities recovered from surfaces in patient rooms where the clean textiles were placed in service [38]. Consistently, one quarter of all airborne bacterial RNA genes recovered from both the textile holding rooms monitored, regardless of season, contained sequences indicative of those found in the Firmicute genus *Staphylococcus*.

With exception to bacterial from the genus *Proprionibacterium*, which accounted for more than one third of the bacterial bioaerosol assemblage in the patient floor textile holding room in the early winter, 16S RNA genes representative of *Staphylococcus* spp. were the most abundant observed in the aerosol of these rooms. In all cases, Actinomycete genes from the genus *Corynebacteria* were present in substantial abundance—between 5 and 15 %—followed by a balance of 16S RNA genes indicative of *Lactobacillus* spp. and *Streptococcus* spp., the sum of which always exceeded 5 % of the total. A pool of between 20 and 30 % of the 16S RNA genes recovered from this environment comprised a conglomerate of genera, each of which was less than 1 % of the overall relative abundance.

With exception to the recovery of a substantial abundance (8 %) of 16S RNA genes from *Micrococcus* spp. in the basement textile storage area, the genes comprising these bacterial bioaerosol assemblages have obvious overlap in the context of the built environment in which they operate given the following conditions: (i) the source aerosols are separated by nine floors and connected by an unventilated conduit which is episodically used; (ii) both textile holding rooms are isolated and have little human intervention; (iii) the textile holding room on the patient floor (origin) is passively ventilated with HEPA-filtered air; and (iv) the terminal textile holding room on the basement floor has no ventilation.

## Discussion

Hospitals have been recently shown to house complex assemblages of airborne and surface-associated microorganisms, many of which likely emanate from patients and health care workers in routine interactions [39]. In general, the levels of airborne microbes found in (many) health care environments are considered to be well below those found in other indoor commercial environments because of specialty building design, materials, and operations (cleaning and HVAC) [40]. The profile of airborne microbial communities encountered in these unique indoor environments is likely different in both quantity and relative abundance on room, hallway, or even floor scales with respect to their commercial or residential counterparts [41].

Socioeconomic drivers have focused increased attention to the characterization and control of nosocomial disease. In this context, relative microbial exposures in health care settings are apportioned between contact surfaces (touch) and aerosol association (inhalation and fomite); the balance of those exposures is currently unknown [18, 42]. What is now known about bioaerosols in this context is that they originate from all persons, regardless of health status [10, 43], as well as the broad variety of service textiles (bedding, uniforms, privacy curtains, etc.) [44, 46], wastes [46, 47], and building materials unique to the modern health care environment [48]. Considering that mandatory personal hygiene (e.g., hand washing) [17] and cleaning practices are up to the most modern standards and assessments, nosocomial diseases remain at alarming levels; this precipitates better characterization of occupational bioaerosol as a last frontier needed for expanding risk assessment in this arena [49].

A substantive fraction of literature, based primarily on culture-based microbial characterization methods, suggests the potential to significantly increase local (bio)aerosol concentrations when handling hospital textiles—even where bagging is employed. The contemporary optical loading and phylogenetic characterization presented in this demonstration study are consistent with this hypothesis. This survey capitalized on a unique opportunity to characterize (bio)aerosols in isolated textile handling rooms, just prior to, and following their commissioning into full hospital service. It is only through isolation—either in the field or in laboratory-based chamber studies—that the magnitude and character of bioaerosol from these sources may be reliably estimated. Toward this practical isolation condition, unique here was the fact that soiled textiles, originally held in a HEPA-filtered patient area, communicated bioaerosol to a terminal storage room through a single conduit with a single commodity—soiled textiles, bagged, or otherwise. Given it was far removed from the source (nine floors), the fact that the terminal holding room is, by design and operational policy, sealed, unventilated, and rarely occupied, the particle characterizations and bacterial assemblages recovered here suggest that soiled textile handling liberates significant quantities of bacterial bioaerosol in the absence of immediate human intervention. With specific respect to linen holding sites and chutes, their design and operation are intended to be under negative pressure with respect to their immediate surroundings, a condition which was episodic in the originating textile holding room monitored here. With the service load of hospital linens, it is not surprising that the dominant bacterial bioaerosol assemblages observed here are found in abundance associated with human hair, skin, and to a lesser degree gut. Given the resolution of the Illumina platform and the most common primers and pipelines used to process bacterial phylogenetic data, the more dominant gene sequences in assemblages observed here are the same as those reported from recent bioaerosol studies of selected wards [41] and isolated classrooms [10]; however, the relative abundances of the dominant genera are markedly different, which may be due to different sampling schemes (dry filters) and sequencing/processing platforms used (e.g., pyrosequencing, QIIME). Although the dominant genera observed contain species that are known nosocomial pathogens, they cannot yet be unambiguously identified in these types of data sets given the resolution of the bacterial DNA primers associated with the most current Illumina platforms.

By concomitantly using light scattering and  fluorescence signatures, bioaerosol is discriminated from those particles that are likely non-biological aerosols when gated to a library of known airborne bacteria and fungi [23, 25]. As with its forensic genetic counterpart, an optical fluorescence approach has also been applied to characterize particle emissions in isolated classrooms [50]. Multi-channel fluorescence appears to provide a surrogate for real-time bioaerosol reporting; this is particularly true for many settings in health care environments, given the low potential for environmental interferences in the relatively clean atmosphere found in filtered air in this (HEPA) and other hospitals.

This study was limited to observations of selected patient and textile holding rooms prior to and following the occupation and operation of a modern hospital outfitted with aerosol mitigation equipment. This demonstration was limited in its isolation power, given that rooms and hallways immediately adjacent to the textile holding room monitored were not concurrently sampled. Nonetheless, the study provides molecular-based evidence for bioaerosol generation of sequestered soiled textiles. However, the resolution of the molecular and optical techniques applied can not be leveraged a source-tracking paradigm to implicate specific patient-pathogen sources.

## Conclusion

Taken together, composite optical particle recognition with bacterial phylogenetics suggests that aerosol partitioning from the routine handling and storage of soiled textiles can contribute to airborne microbial exposures in the health care environment. This approach may inform critical path analyses to better understand the potential impacts of textile and residual waste handling practices and the paths soiled textiles and other medical wastes typically take through hospitals. Changing these residual handling practices in response to detailed spatial and temporal patterns of in situ bioaerosol loads may help reduce nosocomial (aerosol and fomite) exposure potentials, because both the staff and materials involved in hospital residuals management “shed” as they move and can shed where stored. This has implications for both the aerosol transfer of microbes, as well as the spreading of fomites in health care settings.

## Availability of supporting data

The accession numbers of the nucleic acid sequences reported here are filed with NCBI under [GenBank:SRP063419].
